# Neural network assisted high-spatial-resolution polarimetry with non-interleaved chiral metasurfaces

**DOI:** 10.1038/s41377-023-01337-6

**Published:** 2023-12-04

**Authors:** Chen Chen, Xingjian Xiao, Xin Ye, Jiacheng Sun, Jitao Ji, Rongtao Yu, Wange Song, Shining Zhu, Tao Li

**Affiliations:** Nanjing University, National Laboratory of Solid State Microstructures, Key Laboratory of Intelligent Optical Sensing and Manipulations, Jiangsu Key Laboratory of Artificial Functional Materials, College of Engineering and Applied Sciences, 210093 Nanjing, China

**Keywords:** Metamaterials, Imaging and sensing

## Abstract

Polarimetry plays an indispensable role in modern optics. Nevertheless, the current strategies generally suffer from bulky system volume or spatial multiplexing scheme, resulting in limited performances when dealing with inhomogeneous polarizations. Here, we propose a non-interleaved, interferometric method to analyze the polarizations based on a tri-channel chiral metasurface. A deep convolutional neural network is also incorporated to enable fast, robust and accurate polarimetry. Spatially uniform and nonuniform polarizations are both measured through the metasurface experimentally. Distinction between two semblable glasses is also demonstrated. Our strategy features the merits of compactness and high spatial resolution, and would inspire more intriguing design for detecting and sensing.

## Introduction

Amplitude, phase, polarization, and wavelength are fundamental properties of light. Among them, the state of polarization (*SoP*) characterizes the direction in which the electric component of the field oscillates. The analysis and measurement of the *SoP* plays a key role in wide applications from remote sensing^1^ and astronomy^2^ to biology^3,4^ and microscopy^5^ since the light-matter interactions have strong dependences on the *SoP*. Hence, various types of polarization detection (i.e., polarimetry) systems have been developed over the past decades. In general, traditional polarimetry systems can be categorized into division of time and division of space^6^. The former requires rotating polarization elements resulting in long detection time. The latter can be further grouped into division of amplitude, aperture, and focal plane. All of these techniques are equipped with a set of polarizers, waveplates, beam-splitters or filters, making the systems bulky and complex, therefore hinders the future development for miniature and compact optical devices.

Metasurface, a new emerging flat optical device, enables thin and lightweight optical elements with precisely engineered wavefronts^7–17^. Many innovative methods based on metasurfaces for polarimetry have been proposed. Constructing a subwavelength scatterer^18^ or antenna array^19^, on-chip polarimeter can be realized, however, the propagation waveguide for polarization-selective coupling or the free space for directional scattering restricts the application in full local mapping of the inhomogeneous *SoP*. Based on polarization filters and spatial multiplexing scheme^20,21^, full-Stokes polarimetric measurements can be obtained with complex fabrication due to the dual-layer configuration^22–24^. Polarimetry can also be demonstrated with the design of plasmonic meta-gratings^25,26^, yet the reflection/diffraction mode makes it challenging for direct integration on sensors. Similar to the division of focal plane, researchers design a metalens array (usually consisting three to six metalenses) to split and focus light in different polarization bases for estimating the full Stokes vectors^27–31^. However, this spatial interleaved design method still suffers from the trade-off between the detection pixel size (i.e., 3-6 metalenses) and measurement crosstalk, prohibiting them to access high-spatial resolution in polarization analyses. Matrix Fourier optics has also been introduced and applied to the realization of polarimetry^32^, whereas the design needs to be fed into an optimization and the polarization-dependent propagation with different diffraction orders would occupy a substantial volume of space.

Most of the relevant works focused on the intensity measurement of different polarization bases (at least four) to calculate the Stokes vector. Actually, for an arbitrary *SoP*, it can be decomposed into a pair of orthogonal polarization states (e.g., right-hand and left-hand circular polarizations, termed as RCP and LCP) with different amplitudes and phase shifts. If the amplitude contrast and the phase difference can be detected simultaneously, then the *SoP* can be obtained one time without spatial multiplexing.

Here, we propose a new strategy based on a non-interleaved chiral metasurface and neural network assistance to analyze the *SoP* in high spatial resolution. This single chiral metasurface can modulate the co-polarization and two cross-polarizations independently to present the amplitude and phase information. Both spatially uniform and nonuniform polarization states are detected in simulations and experiments, showing very good fidelity. Note that in the spatially nonuniform polarization detection, an inhomogeneous *SoP* beam is generated by a specially designed metasurface, and a neural network is employed to strengthen the detection of *SoPs*. Finally, we demonstrate its applications in daily life for picking out different functional glasses with similar morphology features.

## Results

Stokes parameters defined as *S*_0_ = |*E*_*x*_ | ^2^ + |*E*_*y*_ | ^2^, *S*_1_ = |*E*_*x*_ | ^2^-|*E*_*y*_ | ^2^, *S*_2_ = 2|*E*_*x*_ | |*E*_*y*_|cos*δ*_*xy*_, and *S*_3_ = 2|*E*_*x*_ | |*E*_*y*_|sin*δ*_*xy*_, are widely utilized to represent the *SoP* of light^33^, in which *E*_*x*_ and *E*_*y*_ are the complex amplitudes of the *x* and *y* polarized components, *δ*_*xy*_ is the corresponding phase difference. Based on the Stokes parameters, the ellipticity angle *χ* and the azimuth angle *ψ* for a polarized light can be expressed as

1 $$\begin{array}{c}\chi =\frac{1}{2}\arcsin \frac{2|Ex||Ey|}{E{x}^{2}+E{y}^{2}}\,\sin {\delta }_{xy},\,-\frac{\pi }{4}\le \chi \le \frac{\pi }{4}\\ \psi =\frac{1}{2}\arctan \frac{2|Ex||Ey|}{E{x}^{2}-E{y}^{2}}\,\cos {\delta }_{xy},\,0\le \psi \le \pi \end{array}$$

where 2*χ* and 2*ψ* are also the spherical coordinates on the surface of Poincaré sphere. Under the circular base, these two parameters can be written as

2 $$\begin{array}{l}\chi =\frac{1}{2}\arcsin \frac{{|{E}_{R}|}^{2}-{|{E}_{L}|}^{2}}{{|{E}_{R}|}^{2}+{|{E}_{L}|}^{2}},\,-\frac{\pi }{4}\le \chi \le \frac{\pi }{4}\\ \psi =\frac{1}{2}{\delta }_{cp},\,0\le \psi \le \pi \end{array}$$

in which *E*_*R*_ and *E*_*L*_ are the complex amplitudes of the RCP and LCP components, *δ*_*cp*_ is the phase difference calculated as ${\delta }_{cp}={\varphi }_{L}-{\varphi }_{R}$. Apparently, the *SoP* can be derived with the learning of the amplitude contrast and phase shift of two CP components.

**Fig. 1:**
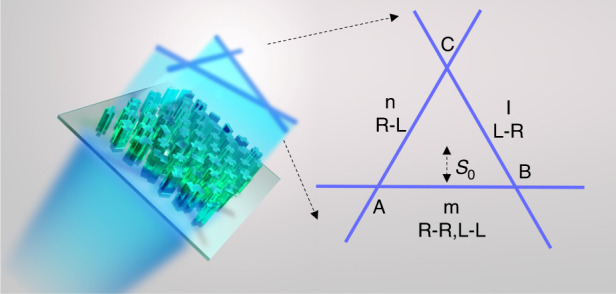
Schematic illustration of the proposed non-interleaved chiral metasurface for polarimetry. *n*, *l*, and *m* are the independent focal lines corresponding to the three channels (RCP to LCP, LCP to RCP, and the co-CP part). *A*, *B* and *C* are the intersections. *s*_0_ corresponds to the off-center distance of the focal lines

To obtain the amplitude and phase information simultaneously, we propose a novel interferometric strategy to analyze the polarization states. As shown in Fig. 1, the designed chiral metasurface generates two focal lines in the case of RCP incidence, *n* is the cross-CP part (RCP to LCP), and *m* is the co-CP part with the point of intersection marked as *A*. While under LCP incidence, similar two focal lines emerge as well, one is the same co-CP component in the position of *m*, and the other is the cross-CP component (LCP to RCP) termed as *l* with the point of intersection marked as *B*. When the *SoP* of incidence is linear or elliptical, all three focal lines appear with a new intersection signed as *C*. With the average intensity distribution of *AC* and *BC*, the amplitude contrast of RCP and LCP components can be obtained. Analyzing the intensity of the points *A* and *B*, for which the electrical fields can be written as

3 $$\begin{array}{c}{E}_{A}={\alpha }_{0}(|{E}_{R}|{e}^{i{\varphi }_{R}}|R\rangle +|{E}_{L}|{e}^{i{\varphi }_{L}}|L\rangle )+{\alpha }_{1}|{E}_{R}|{e}^{i{\varphi }_{R}}|L\rangle \\ {E}_{B}={\alpha }_{0}(|{E}_{R}|{e}^{i{\varphi }_{R}}|R\rangle +|{E}_{L}|{e}^{i{\varphi }_{L}}|L\rangle )+{\alpha }_{2}|{E}_{L}|{e}^{i{\varphi }_{L}}|R\rangle \end{array}$$

where *α*_*0*_, *α*_*1*_, and *α*_*2*_ are the corresponding ratios related to the specific design, the phase difference between RCP and LCP can then be derived as well due to the interferometric effect.

The implementation of the above-mentioned functionalities demands the independent phase modulation for triple polarization channels (RCP to LCP, LCP to RCP, and the co-CP part). Similar independent manipulations of multiple channels (phase or the combination of phase and amplitude) have been demonstrated recently based on a few layers of structures, supercells, adding noise, or optimization methods^34–41^. Here, considering a single planar anisotropic meta-atom, we first analyze the possibility to independently modulate the triple polarization channels. The Jones matrix describing the relation between the input and the output electric field in Cartesian coordinates can be written as

4 $${J}_{xy}=R(-\theta )\frac{\sqrt{2}}{2}\left[\begin{array}{cc}{e}^{{\rm{i}}{\varphi }_{xx}} & {e}^{{\rm{i}}{\varphi }_{xy}}\\ {e}^{{\rm{i}}{\varphi }_{xy}} & {e}^{{\rm{i}}{\varphi }_{yy}}\end{array}\right]R(\theta )$$

in which R(*θ*) is the rotation matrix added to increase the degrees of freedom with Pancharatnam–Berry (PB) phase^42,43^ and the amplitude differences are ignored for a straightforward estimation. After the transformation into a circular base, the Jones matrix expresses as

5 $${J}_{CP}=\frac{\sqrt{2}}{4}\left[\begin{array}{cc}{e}^{i{\varphi }_{xx}}+{e}^{i{\varphi }_{yy}} & ({e}^{i{\varphi }_{xx}}-2i{e}^{i{\varphi }_{xy}}-{e}^{i{\varphi }_{yy}}){e}^{2i\theta }\\ ({e}^{i{\varphi }_{xx}}+2i{e}^{i{\varphi }_{xy}}-{e}^{i{\varphi }_{yy}}){e}^{-2i\theta } & {e}^{i{\varphi }_{xx}}+{e}^{i{\varphi }_{yy}}\end{array}\right]$$

from which it can be concluded that the existence of *φ*_*xy*_ ensures the independent phase manipulation of one co-CP (diagonal term) and two cross-CP (non-diagonal term) light. Thus, the meta-atoms that are about to be utilized should break the mirror symmetry and *n*-fold (*n* > 2) rotational symmetry for the generation of the cross components of electric polarizability (i.e., *e*^i*φxy*^). Inspired by our precious work^44,45^, planar chiral meta-atoms are chosen for the decoupling purpose, and the design principle simplifies to

6 $$\begin{array}{l}\,\,{\varphi }_{co}={\varphi }_{d}\\ {\varphi }_{RL}={\varphi }_{\chi RL}+{\varphi }_{PB}\\ {\varphi }_{LR}={\varphi }_{\chi LR}-{\varphi }_{PB}\end{array}$$

where *φ*_*d*_ is the propagation phase^46^, *φ*_*PB*_ is the PB phase and *φ*_*χRL*_ and *φ*_*χLR*_ refers to the different chiral phase delays for the working LCP light (RCP incidence) and RCP light (LCP incidence) respectively $(\vert\varphi_{\chi RL}\vert \ne \vert \varphi_{\chi LR}\vert)$.

**Fig. 2:**
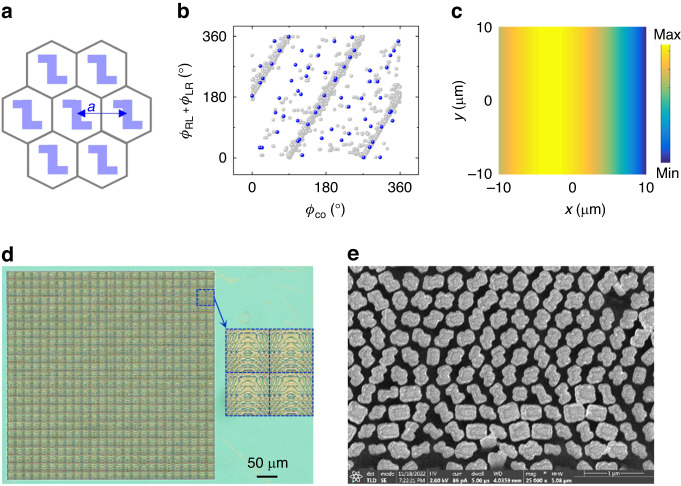
Design and manufactured chiral metasurface. **a** Diagram of the unit cell and the hexagonal lattice. **b** Phase parameter space with the selected ones marked in blue. **c** Co-CP phase distribution of a supercell. **d** Optical microscopy image and the enlarged picture of 2 × 2 supercells (40 μm × 40 μm). **e** Enlarged SEM image showing the meta-atom morphology

For the proof-of-concept, we first perform the simulations (using commercial finite-difference time domain (FDTD) software, Lumerical) to build up a meta-atom library. The designed wavelength is set as 470 nm. The material SiN*x* is chosen to have high transmission in visible light (*n* = 2.032 + 0.0013i) and facilitate the integration with Complementary Metal-Oxide-Semiconductor Transistor (CMOS) technology. As illustrated in Fig. 2a, the lattice is hexagonal with the lattice constant *a* = 360 nm to suppress the high order diffraction. The height of the meta-atom is set as 1.2 μm to cover 0-2π phase shift. Changing the structural sizes of the chiral meta-atoms (the nanorods as particular cases are also included), a data cube including different phase delays of the co- and cross polarizations can be obtained. Figure 2b shows the scattered distribution of *φ*_*RL*_ + *φ*_*LR*_ and *φ*_*co*_, indicating a rich parameter space (gray dots). The *φ*_*co*_ (i.e., *φ*_*d*_) and *φ*_*RL*_ + *φ*_*LR*_ (i.e., *φ*_*χRL*+_*φ*_*χLR*_) is simplified under eight-grade phase approximation. The atoms are chosen by simultaneously satisfying *φ*_*co*_ and *φ*_*RL*_ + *φ*_*LR*_ requirements, which enables the co-polarization and two cross-polarization phase modulations by setting appropriate rotation angles, respectively. Besides, the efficiency of the selected atom is expected to be as high as possible. The blue dots mark the final selected atoms with required phase delays and the average efficiency is about 71% (details can be found in Supplementary Note 1).

In order to realize the functionalities stated above for polarimetry (Fig. 1), the three polarization channels need to acquire the uncorrelated phases as

7 $$\begin{array}{l}\,\,{\varphi }_{co}=\frac{2\pi }{\lambda }\left(\sqrt{{s}_{0}^{2}+{f}^{2}}-\sqrt{{(y+{s}_{0})}^{2}+{f}^{2}}\right)\\ {\varphi }_{RL}=\frac{2\pi }{\lambda }\left(\sqrt{{s}_{0}^{2}+{f}^{2}}-\sqrt{{(-x\sin ({\theta }_{n})+y\cos ({\theta }_{n})+{s}_{0})}^{2}+{f}^{2}}\right)\\ {\varphi }_{LR}=\frac{2\pi }{\lambda }\left(\sqrt{{s}_{0}^{2}+{f}^{2}}-\sqrt{{(-x\sin ({\theta }_{l})+y\cos ({\theta }_{l})+{s}_{0})}^{2}+{f}^{2}}\right)\end{array}$$

where *s*_*0*_ determines the offset distance of the focal line from the center, *f* is the focal length, *θ*_*n*_ and *θ*_*l*_ are the angles between the focal line (*n* and *l*) and the *x* axis (*θ*_*n*_ = 120°, *θ*_*l*_ =-120°). Figure 2c shows the co-CP phase distribution of a single metasurface with dimension of *D* = 20 μm, *s*_*0*_ = 3 μm, and *f* = 25 μm, corresponding to an off-axis focused cylindrical lens, while the other two cross-CP phase distributions are the relatively rotated ones. Considering the requirements of both the spatially uniform and nonuniform *SoP* detections, an array (25 ｘ 25) of such chiral metasurface (termed as a detection pixel) is designed and fabricated. Figure 2d displays its optical microscopy image, and the right panel is the enlarged picture of the area marked by blue dotted line with four detection pixels (2 × 2) and sizes of 40 μm × 40 μm. The partial scanning electron microscopy (SEM) image is also illustrated in Fig. 2e, indicating the basic morphology maintained.

**Fig. 3:**
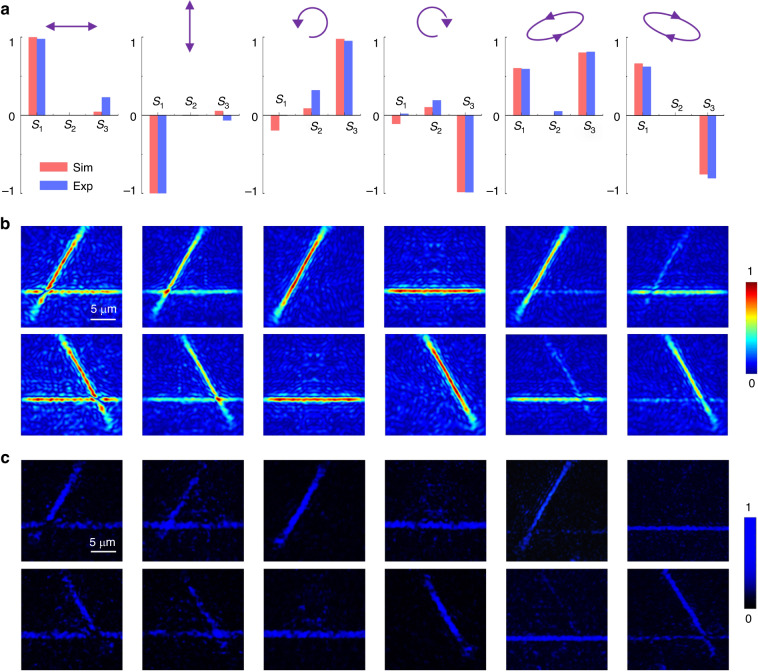
Polarimetry results for different uniform polarizations. **a** Calculated **S** parameters from simulation and experiment results. **b** The focal plane electrical field profiles with LCP bias (top panel) and RCP bias (bottom panel) in simulation. **c** The focal plane intensity profiles for LCP light (top panel) and RCP light (bottom panel) in experiments

To validate the polarimetry function, we first perform simulations and experiments with different uniform polarization incidences. Figure 3 show the results for six polarizations including two linear polarizations (*x* with **S** = (1,0,0),and *y* with **S** = (−1,0,0)), two circular polarizations (LCP with **S** = (0,0,−1), and RCP with **S** = (0,0,1)) and two elliptic polarizations (**S** = (0.64,0,0.77), and **S** = (0.64,0,-0.77)). The full-wave simulation results (performed by Lumerical software) are illustrated in Fig. 3b. The focal lines are not ideally homogeneous and the background noise emerges due to the invalid of local periodic approximation^47,48^. Thus, in addition to the ratios in Eq.3, circular polarization analysis (extracting the field profiles under two CP biases in simulations and adding two CP filters in experiments) are further applied to improve the accuracy of polarization calculation and analysis. The top panel in Fig. 3b corresponds to LCP components and the bottom is RCP. In the first column (*x* polarization), line *n* and *m* appear in the LCP bias (top), corresponding to the co-CP part of the LCP component and the cross-CP part of the RCP component. Destructive interference occurs at the point A due to the π difference between the RCP and LCP components, corresponding to *δ*_*cp*_ = 0 (the π difference is the original phase shift between the RCP and LCP components which is considered in all cases). As for RCP bias (bottom), line *l* and *m* appear and the point B also disappears due to the destructive interference. In the second column (*y* polarization), line *n* and *m* appear in the LCP bias (top) and line *l* and *m* appear in the RCP bias (bottom) similarly, yet the point A and B are both enhanced about twofold intensity, indicating constructive interference with *δ*_*cp*_ = π. For CP light incidences, things get easier, only one line appears in the different biases, as shown in the third and fourth column. With elliptic polarization incidence, the situation gets complicated, two lines appear with distinct intensity contrast in both LCP and RCP biases. By carefully calculating the amplitude contrast and the intensities of points A and B, the ellipticity angle *χ* and the azimuth angle *ψ* can be obtained and the **S** parameters are further derived, shown in Fig. 3a. More results of other uniform polarizations are shown in Supplementary Note 2.

Based on about 34 different polarization incidences, the average transmission of the metasurface defined by the transmitted power divided by the incident power is about 80%. The average diffraction efficiency defined by the power of the focal line within three times full-widths at half maximum (FWHM) divided by the transmitted co-polarized light is calculated ~70%. Thus, the total efficiency defined by the average transmission multiplied by the average diffraction efficiency is calculated as about 56%, indicating potential applications in real-world scenarios. The details can be found in Supplementary Note 2.

In experiments, the metasurface was illuminated by a white-light laser (Fianium Super-continuum, 4 W) with a 470 nm filter with a bandwidth of 10 nm. A polarizer (Thorlabs, WP25L-VIS) and a quarter-wave plate (Thorlabs, AQWP05M-600) were used to generate different polarization incidences. Then the focal plane was collected to the image sensor through an objective (NA = 0.5) with a clearer vision of details. Figure 3c illustrates the captured images (one detection pixel) with different commercial CP filters (top panel showing LCP component and bottom showing RCP component). The results are consistent with the simulations while with different intensity ratios due to the fabrication errors. The calculated **S** parameters are also shown in Fig. 3a, close to the theoretical values.

**Fig. 4:**
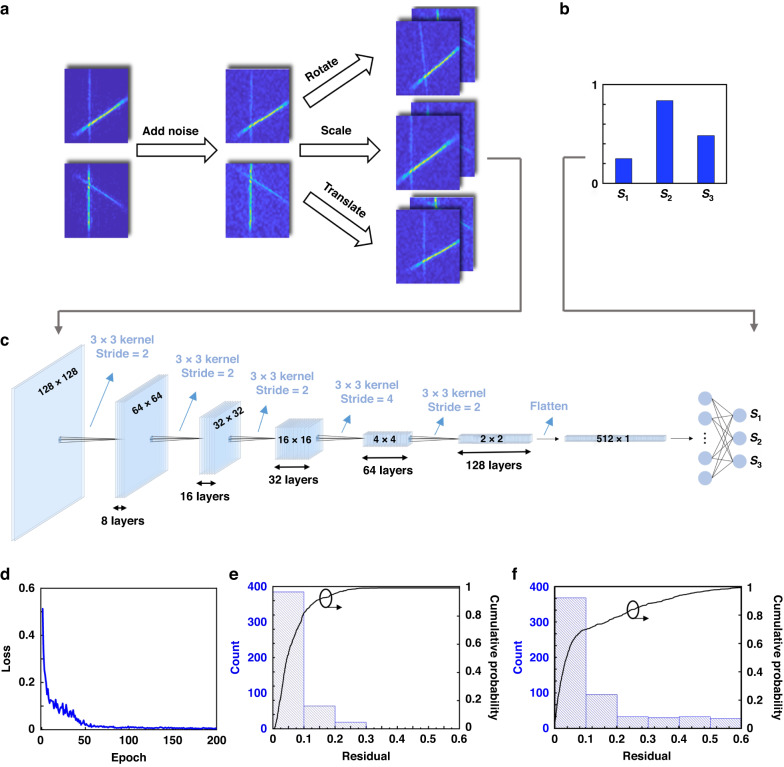
Neural network architecture for the polarimetry. **a** The input of the neural network with data augmentation. **b** The corresponding real **S** parameters of the input data in **a**. **c** The structure and parameters of the CNN. **d** The distribution of MSE with respect to the train epoch. The statistics distribution and cumulative probability distribution of the prediction deviation for the test set with spatial resolution of (**e**) 20 μm, (**f**) 10 μm

To enable the polarimetry as precisely as possible, we need to locate the point A and B carefully, which inevitably brings the inconvenience when the experimental setup is changed or the image patterns are distributed in different positions of the sensors. To address these issues, we introduced a deep learning framework^49–53^ to assist with polarization detection. Such a deep learning framework involves training neural networks to learn the relations between the detected images and the corresponding S parameters. The construction of neural-assisted polarization detection process is divided into two steps: preparation of training data and the training process of the neural network, as is shown in Fig. 4a–c. In the first step, to obtain a sufficient amount of training data within a limited time and enhance the robustness of the entire system as well, we used data augmentation to process the experimental and simulated data, such as including noises, performing image translation, rotation, scaling and other techniques, as is shown in Fig. 4a. These operations can help the neural network analysis mitigate the influence from the fluctuation of surrounding environment to some extent, and improve the potential for practical applications. After one image augmentation operation, we could obtain two 128 × 128 images, corresponding to focal plane intensity profiles with LCP bias and RCP bias, respectively. The combination of these two images result in a dual-channel three-dimensional image (with a size equal to 128 × 128 × 2), which serves as an input to the neural network. The output of the neural network is a 1 × 3 vector representing the corresponding Stokes parameters (S_1_, S_2_, S_3_) of the input data, as is shown in Fig. 4b. After data augmentation, we ultimately obtained 8500 sets of training data and 585 sets of test data. Such the amount of training data is sufficient for dealing with this kind of particular application scenario because all the pictures share a similar style. The test data without overlapping with training data was used to evaluate the trained network’s performance.

In the second step, we constructed a deep convolutional neural network (CNN), whose structure and parameters are shown in Fig. 4c. Each input data was processed through 6 consecutive convolutional layers, during which hidden features in the intensity profiles were extracted and calculated. After flattening the output of the CNNs into a 1D vector (512 × 1) and passing through a fully connected layers, the predicted Stokes parameters was generated. Throughout the network, a ReLU activation function was applied to each layer except for the last one, for which there was no activation function^52^. After constructing the neural network, we trained this network based on the previously obtained 8500 sets of training data. The loss function used in the training process is the mean square error (MSE)^51^, which is defined as follows:

8 $$MSE=\frac{1}{3}\mathop{\sum }\limits_{i=1}^{3}{({S}_{i}^{{\rm{real}}}-{S}_{i}^{{\rm{pre}}})}^{2}$$

where *S*^*real*^ is the real **S** parameters for the input data, while *S*^*pre*^ is the predicted one generated by the CNN. The train epochs were set as 200, and the distribution of MSE with respect to the train epoch was shown in Fig. 4d. When the training was completed, the MSE was 0.003 for the training set, which corresponds to an average prediction standard deviation (sqrt (MSE)) of 0.055 for each **S** parameter. Then, we demonstrated the accuracy of the well-trained CNN by putting all test sets into it, and calculated the corresponding deviation. Figure 4e shows the statistics distribution as well as the cumulative probability of the prediction deviation for the test set, where the average deviation for each **S** parameter is no more than 0.1 for 85% of the test set and the average deviation for all the test data is about 0.06, indicating a very good fidelity. The performance on the test set demonstrates that the CNN model has good predictive ability and is robust because it works well even when input images have been translated, rotated, or scaled in different length, angle, or ratio, respectively. With the help of the CNN model, there is no need to locate the points A and B for every image precisely, which can help us to calculate out the polarization state of the light in very short time (< 0.05 s for each image).

Moreover, the dimension of the single metasurface can be further reduced to 10 μm with *s*_0_ = 2 μm and *f* = 12 μm, corresponding to a higher spatial resolution. Due to the limitation of our current experimental conditions, only simulations under different incidences are performed to construct the foundation of the training and test data. The statistics distribution and the cumulative probability of the prediction deviation for the new test set are shown in Fig. 4f, in which the average deviation for all the test set is about 0.108, and the percentage of the average deviation no more than 0.1 is 70%, also indicating reliable predictions.

**Fig. 5:**
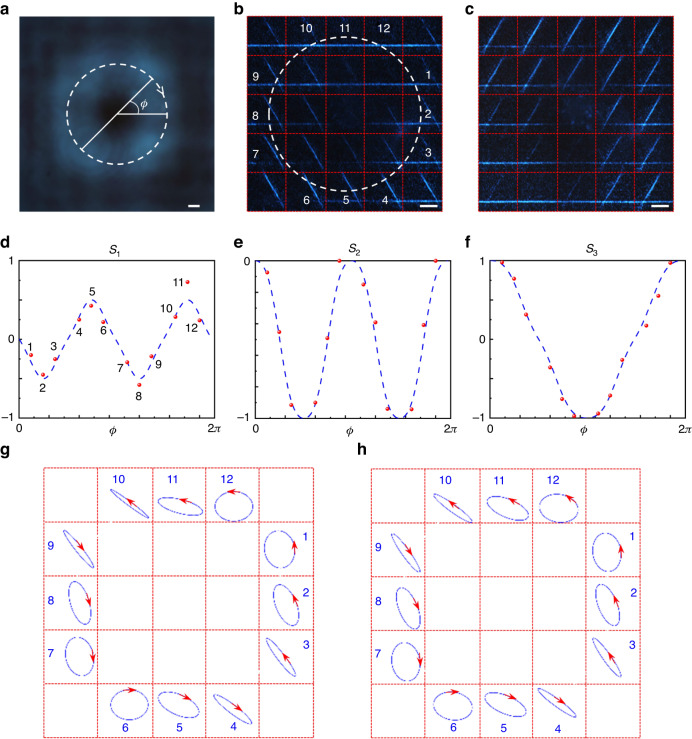
Experimental results for spatially nonuniform SoP analysis. **a** Intensity distributions of the spatially nonuniform polarized incident beam. *φ* is the azimuth angle with respective to the drawn diameter. Focal plane intensity profiles of the chiral metasurface array with LCP (**b**) and RCP (**c**) bias. The scale bar is 10 μm. **d**–**f** Analysis of the Stokes parameters with the assistance of the proposed neural network. The number of the red points is in accordance with the position and azimuth angle in **b** and **a**. The dotted blue lines are the designed **S** parameters. **g** Schematics of the polarization distribution according to the measured **S** parameters. **h** Schematics of the designed polarization distribution

Furthermore, in order to demonstrate the spatially nonuniform *SoP* analysis capability, a specifically designed PB-metasurface with diameter of *D* = 200 μm was fabricated to generate a beam with inhomogeneous polarization states for the measurement (details can be found in Supplementary Note 3). In experiments, the linearly polarized laser beam with wavelength of 470 nm was first enlarged by a beam expander (see Fig. S7), then passed through the PB-metasurface and formed a vector beam by acquiring different phase shifts with different biases (Fig. 5a). Then this vector beam illuminated on the chiral metasurface array and was focused to the image plane with different patterns in each detection pixel. An objective accompanying by a CP filter was further utilized to transfer the image to a CMOS sensor. Figures 5b, c displays the focal plane intensity profiles with LCP bias and RCP bias, respectively. We first align the center of the beam pictures without and with passing through the metasurface polarimetry, corresponding to Fig. 5a, b (or 5c). Then extracting the distance between adjacent intersections as the mesh size to draw the dividing network. The dotted line marks the beam size, in line with the one in Fig. 5a. The region of interest marked in numbers corresponds to the middle of the beam. Assisted by the neural network proposed in Fig. 4, we can locally map the Stokes parameters easily, as shown in Fig. 5d–f. The number of the red points in the figures corresponds to the positions in Fig. 5b, c with different azimuth angles. The dotted blue line shows the designed continuously variable *SoP*, in high accordance with the neural network assisted mapping. According to the measured Stokes parameters, the schematics of polarization distribution are drawn in Fig. 5g for an intuitive view, which shows very good coincidence with the designed one (Fig. 5h). The polarization distribution is more complicated than ideal situation (radially-polarized vector beam) due to the partial circular polarization conversion, however, such complexity happens to prove the polarimetry capability of the proposed neural network assisted chiral metasurface.

**Fig. 6:**
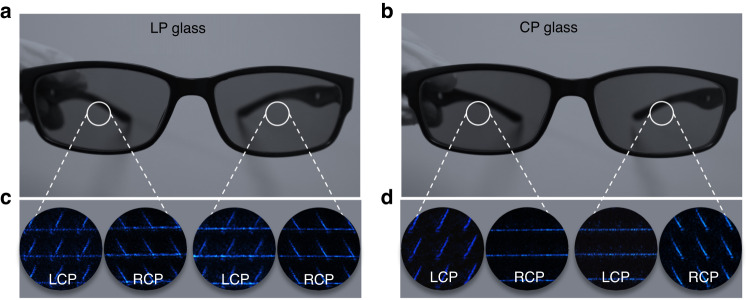
Experimental results glasses distinction. Photos of two pair of glasses with similar morphology features while different functions, **a** is linear polarized glasses, **b** is circular polarized glasses, **c** and **d** are the relevant measurement results

Objects that have similar appearances but with different materials or functions are ubiquitous in our daily lives. Here in the final part, the non-interleaved chiral metasurfaces were implemented to distinguish two semblable glasses. As shown in Fig. 6a, b, these glasses have similar morphology features that we cannot differentiate from each other with our naked eyes. Yet enabled by the chiral metasurfaces, the specific function of each eyeglass can be discovered. In experiments, the generated light without defined polarization is first filtered by the wavelength of 470 nm and transmits through a beam expander to illuminate the pieces of glasses, respectively. The transmitted light further passes through the chiral metasurfaces, and is captured by an objective and a CMOS sensor (Fig. S8). Figure 6c, d illustrate the intensity profiles under different CP biases, from which the differences of the glasses can be easily distinguished. In Fig. 6c, the left and the right glass exhibit the same linear *SoP* patterns with the metasurface when light passes through them, indicating the feature of linear polarization for sunscreen purpose (termed as LP glass). While in Fig. 6d, the left and right glass shows totally different circular *SoP* patterns, revealing the feature of RCP and LCP for 3D display purpose (termed as CP glass).

## Discussion

To summarize, we present a novel interferometric polarimetry method based on non-interleaved chiral metasurfaces. Different uniform polarizations are measured both in simulations and experiments. Furthermore, incorporated with a deep convolutional neural network, spatially nonuniform polarizations are experimentally analyzed and mapped with resolution of 20 μm in a highly accurate, quick, and robust way. The spatial mapping resolution can also be increased to 10 μm assisted by the neural work. Objects with similar morphology features while different polarization characteristics can also be easily distinguished through the metasurface.

The proposed non-interleaved design is a clear departure from conventional ones, and can enable the polarimetry with higher spatial resolution. Normally, the dimension of the single metasurface cannot be further reduced to a very small value, in which the insufficient sampling would result in inaccuracy, crosstalk, or even invalidation of the measurements. Such embarrassment can be alleviated by utilizing neural network. Although with a slight degraded performance as the dimension decreases, the incorporation of the neural network is still promising to access to higher space resolution with merits of quickness and robustness. While for mixed polarization scenarios with incoherent light, the proposed scheme is hard to handle due to design principle based on interferometric effect. Note that due to the mismatch between the focal length and the working distance of our CMOS sensor, we did not directly mount the metasurface on the sensor to build a compact prototype. Nevertheless, the direct transmission mode guarantees the further integration with sensors to realize miniature and compact devices in the near future. The proposed scheme can undoubtedly extend to other spectral bands and would shines in the situation with high spatial resolution requirements for polarimetry.

## Materials and methods

The metasurface was prepared with a combination process of electron-beam lithography (EBL) and reactive ion etching (RIE) process. First, the SiN*x* layer was deposited on the fused-silica substrate using the plasma-enhanced chemical vapor deposition (PECVD) to a thickness of 1200 nm. Then PMMA A4 resist film with thickness of 200 nm was spin-coated onto the substrate and baked at 170 °C for 5 min. Next, a 42 nm thick layer of a water-soluble conductive polymer (AR-PC 5090) was spin-coated on the resist for the dissipation of E-beam charges. The device pattern was written on an electron beam resist using E-beam writer (Elionix, ELS-F125). The conductive polymer was then dissolved in water and the resist was developed in a resist developer solution. An electron beam evaporated chromium layer was used to reverse the generated pattern with a lift-off process, and was then used as a hard mask for dry etching the silicon nitride layer. The dry etching was performed in a mixture of CHF3 and SF6 plasmas using an inductively coupled plasma reactive ion etching process (Oxford Instruments, PlasmaPro100 Cobra300). Finally, the chromium layer was removed by the stripping solution (Ceric ammonium nitrate).

### Supplementary information

Supplementary Material for Neural network assisted high-spatial-resolution polarimetry with non-interleaved chiral metasurfaces

## Data Availability

The source data are available from the corresponding author upon request. All data needed to evaluate the conclusion are present in the manuscript and/or the Supplementary Material.
