# Supplementary material for: Neural network assisted high-spatial-resolution polarimetry with non-interleaved chiral metasurfaces
Source: Light Sci Appl. 2023 Dec 4;12:288. doi: 10.1038/s41377-023-01337-6 (PMC10694149; doi:10.1038/s41377-023-01337-6)
Supplement: Supplementary file 1 — Supplementary Material for Neural network assisted high-spatial-resolution polarimetry with non-interleaved chiral metasurfaces [file 41377_2023_1337_MOESM1_ESM.docx]

**Supplementary Material for**
Neural network assisted high-spatial-resolution polarimetry with non-interleaved chiral metasurfaces

Chen Chen,**^†^** Xingjian Xiao**^†^**, Xin Ye, Jiacheng Sun, Jitao Ji, Rongtao Yu, Wange Song, Shining Zhu, and Tao Li^*^

*Nanjing University, National Laboratory of Solid State Microstructures, Key Laboratory of Intelligent Optical Sensing and Manipulations, Jiangsu Key Laboratory of Artificial Functional Materials, College of Engineering and Applied Sciences, Nanjing, 210093, China.*

*†These authors contributed equally to this paper.*

**Corresponding authors. Email:* [*taoli@nju.edu.cn*](mailto:taoli@nju.edu.cn)

**Supplementary Note 1: Details of the chiral meta-atoms.**

**Supplementary Note 2: More results of the uniform polarizations.**

**Supplementary Note 3: Details of the PB-metasurface for generating vector beam.**

**Supplementary Note 1: Details of the chiral meta-atoms.**

As shown in Fig. S1, the chiral meta-atom is arranged in hexagonal lattice with several variable geometric parameters, including the length (*l*_1_) and width (*w*_1_) of the middle rod, the arm length (*w*_2_) and width (*l*_2_) on each side, and the spacing between the rod and arm (*d*). Commercial finite-difference time domain (FDTD) software, Lumerical, is utilized to simulate the phase shifts of co-polarized and cross-polarized channels of the chiral meta-atoms. Perfectly matched layer (PML) is utilized as boundary condition along the propagation direction of plane-wave source while periodic boundary conditions are applied along all the in-plane directions. Finally, 64 chiral meta-atoms are selected from the simulated atom library to obtain the required phase delays under eight grade phase approximation (as marked with blue dots in Fig. 2b), the specific geometric parameters with relative phase shifts are listed in Table S1, and the transmission distribution is shown in Fig. S2.


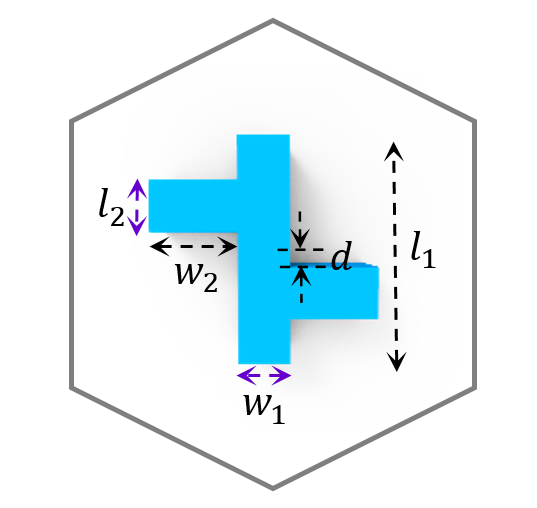


**Figure S1**. Top view of a chiral meta-atom and the variable geometric parameters.

**Table S1. Details of the selected 64 meta-atoms.**

| **W1**  **(nm)** | **L1**  **(nm)** | **W2**  **(nm)** | **D**  **(nm)** | **T** | **φ_RR_**  **(°)** | **φ_χRL_**  **(°)** | **φ_χLR_**  **(°)** | **φ_LL_**  **(°)** |
| --- | --- | --- | --- | --- | --- | --- | --- | --- |
| 110 | 230 | 100 | -55 | 0.51 | 167 | -169 | -169 | 167 |
| 110 | 260 | 100 | -80 | 0.48 | 179 | -94 | 120 | 176 |
| 120 | 290 | 60 | -35 | 0.54 | 175 | 80 | -4 | 164 |
| 70 | 200 | 40 | -100 | 0.97 | 160 | 34 | 106 | 160 |
| 60 | 280 | 40 | -30 | 0.97 | 177 | 87 | 87 | 177 |
| 60 | 290 | 60 | -95 | 0.97 | -158 | 94 | 130 | -159 |
| 70 | 270 | 40 | -15 | 0.85 | -163 | 138 | 111 | -148 |
| 60 | 250 | 70 | -45 | 0.55 | -158 | 137 | -164 | -158 |
| 70 | 290 | 70 | 35 | 0.73 | -118 | -158 | 144 | -117 |
| 260 | 240 | 0 | 0 | 0.34 | -156 | -149 | -149 | -156 |
| 270 | 180 | 0 | 0 | 0.45 | -155 | -129 | -129 | -155 |
| 190 | 280 | 0 | 0 | 0.47 | -126 | 62 | 62 | -126 |
| 280 | 210 | 0 | 0 | 0.58 | -129 | -84 | -84 | -129 |
| 70 | 230 | 60 | 45 | 0.97 | -149 | 162 | 81 | -149 |
| 70 | 260 | 40 | -30 | 0.97 | -138 | 136 | 131 | -138 |
| 70 | 260 | 50 | 0 | 0.99 | -121 | 171 | 130 | -119 |
| 80 | 260 | 40 | -40 | 0.99 | -90 | -180 | -180 | -90 |
| 80 | 250 | 70 | -125 | 0.96 | -77 | 151 | -115 | -70 |
| 90 | 210 | 40 | -25 | 0.66 | -79 | -112 | -167 | -82 |
| 70 | 260 | 100 | 20 | 0.50 | -79 | -58 | -148 | -82 |
| 80 | 210 | 60 | -35 | 0.49 | -85 | -32 | -135 | -85 |
| 80 | 200 | 60 | -30 | 0.62 | -107 | 4 | -152 | -108 |
| 70 | 280 | 70 | 70 | 0.52 | -102 | -155 | 89 | -149 |
| 80 | 220 | 60 | -110 | 0.99 | -103 | 117 | -143 | -103 |
| 70 | 240 | 100 | -40 | 0.65 | -53 | 44 | -22 | -55 |
| 80 | 190 | 100 | -95 | 0.97 | -58 | 104 | -48 | -66 |
| 220 | 130 | 0 | 0 | 0.96 | -47 | 44 | 44 | -47 |
| 90 | 230 | 70 | -115 | 0.95 | -31 | -177 | -60 | -28 |
| 80 | 200 | 80 | -30 | 0.56 | -55 | 52 | 113 | -50 |
| 230 | 140 | 0 | 0 | 0.71 | -31 | 111 | 111 | -31 |
| 90 | 280 | 50 | -30 | 0.51 | -30 | -41 | -59 | -18 |
| 70 | 250 | 90 | -25 | 0.46 | -52 | 65 | -119 | -45 |
| 100 | 240 | 50 | -80 | 0.50 | 6 | -51 | 33 | 6 |
| 80 | 270 | 70 | -25 | 0.51 | 15 | -131 | 162 | 19 |
| 80 | 220 | 0 | 0 | 0.88 | 21 | 57 | 57 | 21 |
| 90 | 180 | 100 | 0 | 0.95 | -14 | 15 | 130 | -23 |
| 90 | 280 | 50 | -90 | 0.95 | -1 | -114 | -70 | -7 |
| 100 | 240 | 60 | -120 | 0.95 | 13 | -129 | -20 | 15 |
| 90 | 270 | 60 | -65 | 0.72 | 14 | -65 | -28 | 45 |
| 280 | 270 | 0 | 0 | 0.48 | -17 | -15 | -15 | -17 |
| 90 | 240 | 90 | -30 | 0.50 | 32 | 114 | -103 | 33 |
| 90 | 240 | 100 | -40 | 0.57 | 62 | 172 | -138 | 73 |
| 90 | 200 | 80 | -60 | 0.48 | 30 | 79 | -10 | 32 |
| 100 | 250 | 40 | -45 | 0.50 | 50 | -99 | -108 | 51 |
| 100 | 270 | 40 | -45 | 0.72 | 27 | -79 | -87 | 32 |
| 240 | 90 | 0 | 0 | 0.86 | 31 | -70 | -70 | 31 |
| 100 | 280 | 60 | 40 | 0.96 | 44 | -6 | -85 | 45 |
| 110 | 260 | 50 | 20 | 0.96 | 60 | 9 | -73 | 59 |
| 120 | 270 | 40 | 15 | 0.91 | 94 | 31 | -34 | 95 |
| 50 | 230 | 40 | -75 | 0.70 | 81 | 8 | 40 | 81 |
| 100 | 260 | 80 | -50 | 0.39 | 88 | -132 | -132 | 88 |
| 60 | 180 | 60 | 30 | 0.63 | 112 | 120 | 3 | 112 |
| 90 | 290 | 70 | -25 | 0.48 | 78 | -53 | -133 | 82 |
| 110 | 250 | 50 | -25 | 0.52 | 95 | -21 | -110 | 97 |
| 100 | 250 | 80 | -85 | 0.74 | 72 | -116 | 46 | 74 |
| 110 | 280 | 50 | -140 | 0.97 | 77 | -51 | 18 | 73 |
| 120 | 280 | 40 | -120 | 0.83 | 131 | -23 | 42 | 99 |
| 50 | 280 | 40 | -140 | 0.99 | 120 | 13 | 46 | 119 |
| 60 | 230 | 40 | 55 | 0.98 | 135 | 69 | 21 | 136 |
| 60 | 240 | 50 | 60 | 0.98 | 150 | 88 | 36 | 155 |
| 200 | 100 | 0 | 0 | 0.57 | 130 | -95 | -95 | 130 |
| 190 | 110 | 0 | 0 | 0.58 | 150 | -75 | -75 | 150 |
| 60 | 270 | 50 | -35 | 0.51 | 134 | 127 | 132 | 133 |
| 120 | 240 | 60 | -120 | 0.57 | 134 | -79 | 45 | 138 |


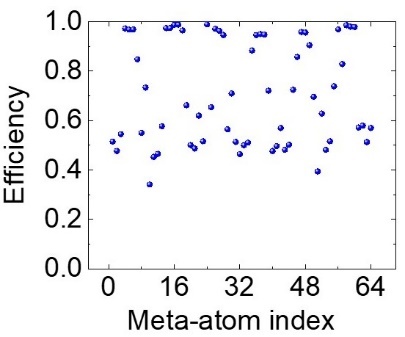


**Figure S2**. Efficiency (average value under both RCP incidence and LCP incidence) distribution of the selected meta-atoms.

**Supplementary Note 2: More results of the uniform polarizations.**

In addition to the results illustrated in Fig. 3, other uniform polarizations are also analyzed both in simulations and experiments. As partially displayed in Fig. S3, the sequentially presented polarizations have S parameters as (0.34,0,-0.94), (0.87,0,-0.5), (0.87,0,0.5), (0.34,0,0.94), (0.5,0.87,0), and (-0.5,0.87,0). The calculated **S** parameters from simulation and experiment results (Fig. S3a) are both close to the theoretical values, showing the powerful polarimetry capabilities of the non-interleaved chiral metasurface.


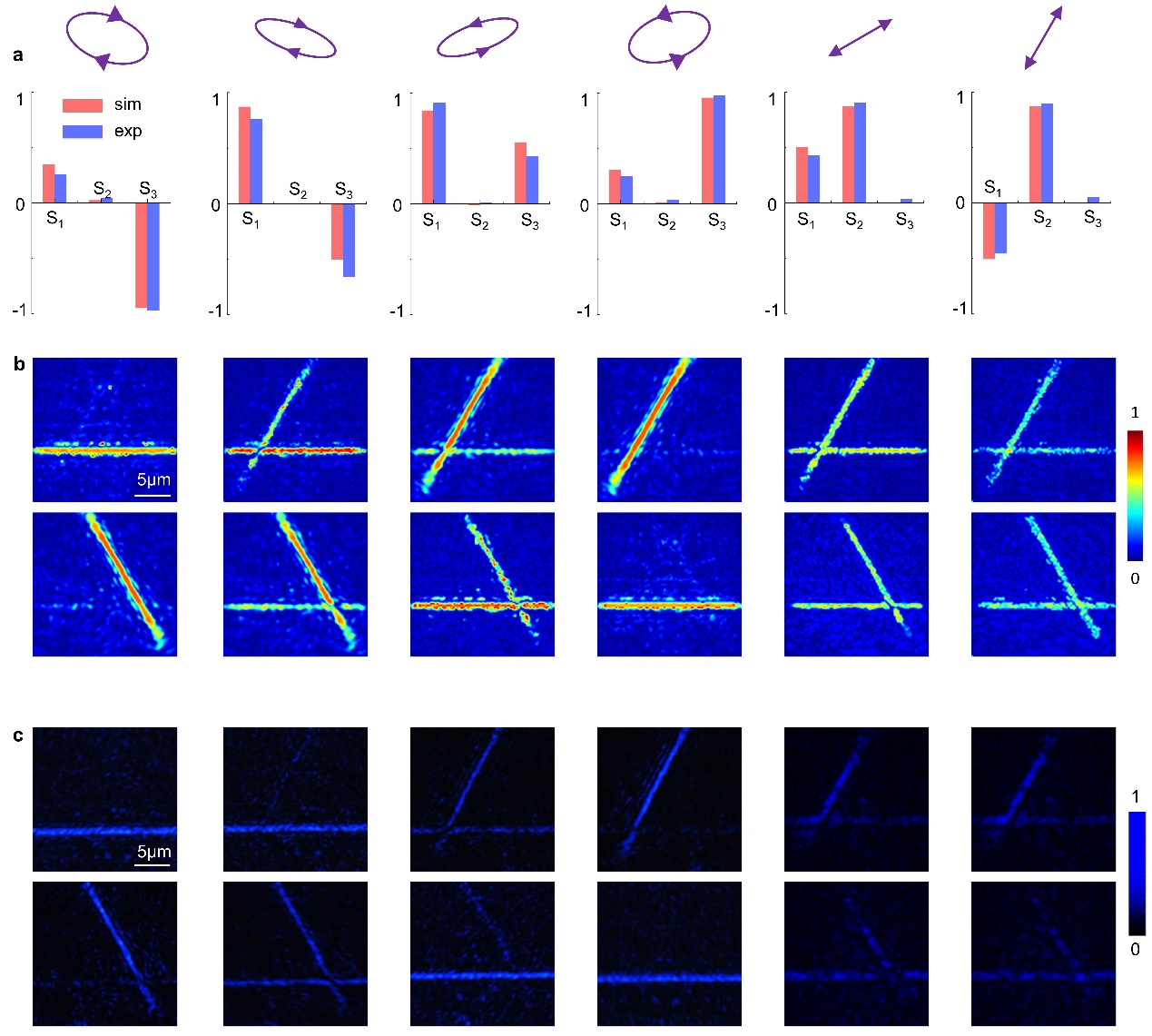


**Figure S3**. More polarimetry results for different uniform polarizations. **a** Calculated **S** parameters from simulation and experiment results. **b** The focal plane intensity profiles with RCP bias (top panel) and LCP bias (bottom panel) in simulation. **c** The focal plane intensity profiles with RCP bias (top panel) and LCP bias filter (bottom panel) in experiments.

As for the metasurface efficiency, the simulated transmission distribution under 34 different incident cases is shown in Fig. S4a with almost negligible differences. The diffraction efficiency distribution is shown in Fig. S4b with a small range of fluctuation due to the different responses of the polarization-dependent chiral atoms.


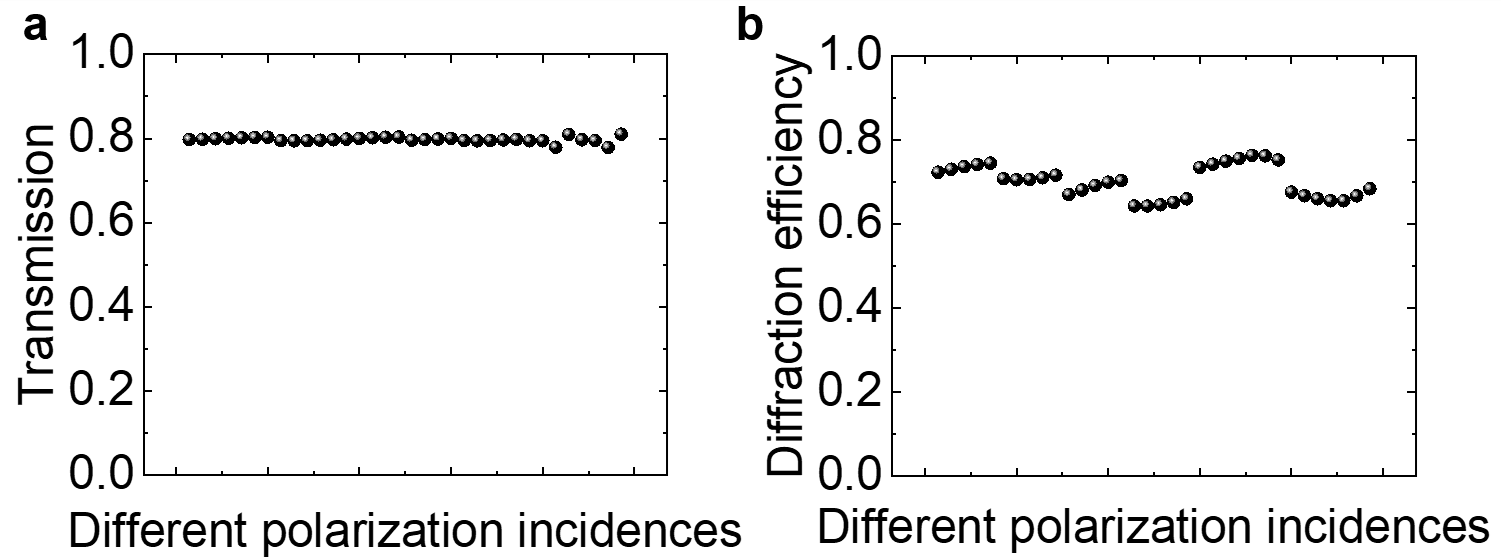


**Figure S4**. **a** Transmission, **b** Diffraction efficiency distribution of the metasurface with different polarization incidences.

**Supplementary Note 3: Details of the PB-metasurface for generating vector beam.**

A PB-phase metasurface was designed to form a vortex beam with the phase distribution given by , in which *l* is the topological charge and set as 1 for one kind of CP light. Certainly, for the other CP light, the vortex beam will have an opposite topological charge as -1. Thus, when the PB metasurface is illuminated by a LP light, it will generate a vector beam with the interference superposition effect.

The specific parameters of the meta-atom incoporated with PB phase modulation is *p*=300 nm, *h*=1000 nm, *w*=95 nm, and *l*=240 nm. The perspective view of the single meta-atom is displayed in Fig. S5a. The optical miscroscopy image of the fabricated metasurface with diameter of 200 μm is shown in Fig. S5b. The fabrication process is similar to that descripted in Methods. Ideally, if the polarization conversion efficiency is 100%, then the superposed light would be radially-polarized-like vector beam. While the fabricated meta-atoms can hardly reach the 100% polarization conversion efficicency due to the fabrication errors and the invalid of the local periodic approximation [1, 2]. Thus, the final interferenced beam would have an inhomogeneous distribution.


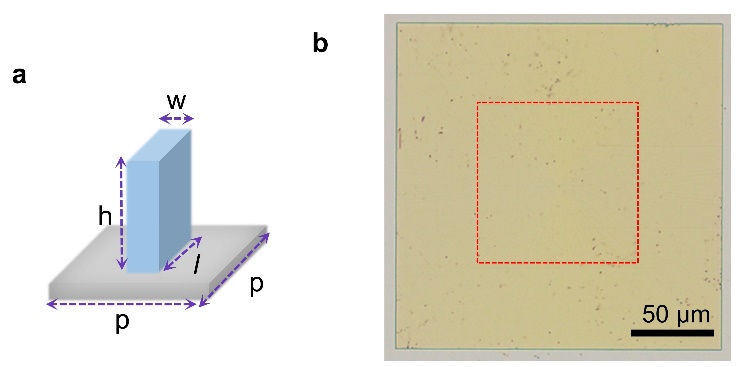


**Figure S5**. PB metasurafce design and image. **a** The perspective view of the meta-atom unit cell of the PB metasurface. **b** The optical miscroscopy image of the fabricated PB metasurface.

In experiments, the polarization conversion efficiency is measured about 50%. With linear polarized (LP) light incidence, the transmitted light is then equivalent to the superposition of a LP beam and a radially-polarized vector beam. Thus, we can decompose the total electrical field to three parts based on RCP, LCP, and LP (here is 135° specifically) light respectively. These three parts all share the same amplitude E_0_ with each phase distribution shown in Figs. S6a-c. The RCP and LCP parts sharing opposite topological charges can interfere together as the radially-polarized vector beam, while the LP (135°) light resulted from the co-CP components has uniform phase distribution. Based on the relative electrical field distribution, the corresponding S parameters of each pixel area (pixel size =20 μm) are illustrated in Figs. S6d-f. Figure S6g further shows the polarization map for an intuitive view with the handness referring to S3 (Fig. S6f), in which the positive values correspond to right-handed properties with anti-clock rotations. The marked regions are the selected ones in the manuscript.


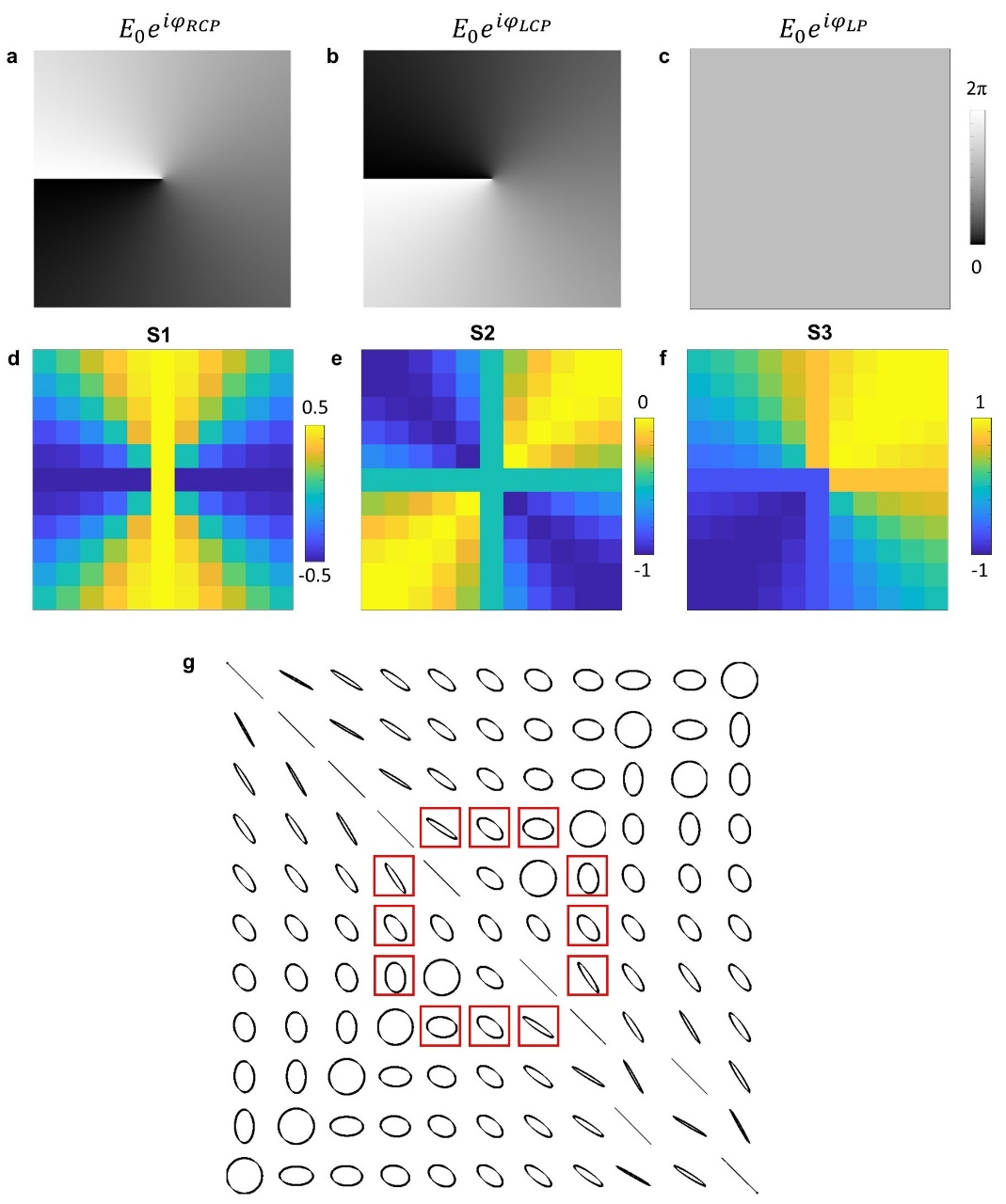


**Figure S6**. Electrical field distribution and polarization maps of the experimental PB-metasurface. Phase distribution of the decomposed **a** RCP light, **b** LCP light, and **c** 135° light. All the amplitude are nearly the same. S parameters distribution as **d** is S1, **e** is S2, and **f** is S3, the pixel size is 20 μm. **g** The polarization map with marked region corresponding to the results in the manuscript.


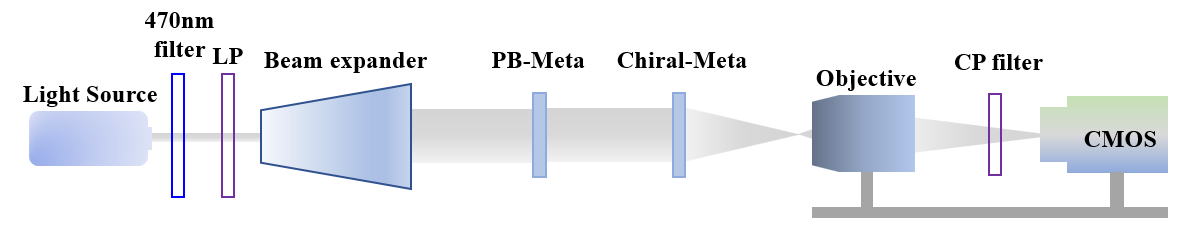


**Figure S7**. Experimental setup scheme for the polarimetry of the vector beam generated by the PB-metasurface.


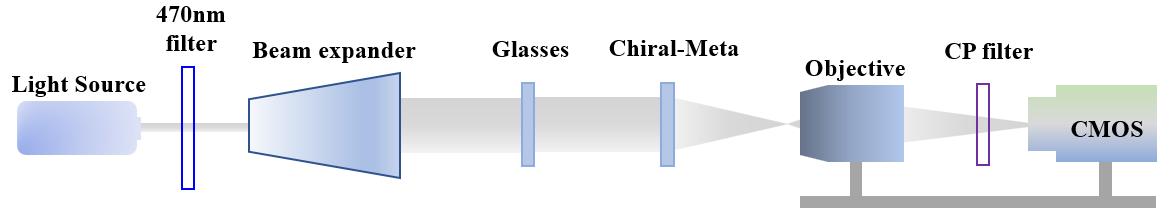


**Figure S8**. Experimental setup diagram for the glasses polarization analysis.

**References**

[1] Pestourie, R. et al. Inverse design of large-area metasurfaces. *Opt. Express* **26**, 33732-33747 (2018).

[2] Arbabi, A. et al*.* Increasing efficiency of high numerical aperture metasurfaces using the grating averaging technique. *Sci. Rep.* **10**, 7124 (2020).
